# Digitally Guided Multidisciplinary Rehabilitation of a Partially Edentulous Patient Using Implant-Supported Prostheses: A Case Report

**DOI:** 10.7759/cureus.86824

**Published:** 2025-06-26

**Authors:** Chaitali Nandedkar, Sunil Ronad, Girija Dodamani, Arun Dodamani, Priyadarshani Pawar

**Affiliations:** 1 Department of Prosthodontics, Jawahar Medical Foundation's Annasaheb Chudaman Patil Memorial Dental College, Dhule, IND; 2 Department of Public Health Dentistry, Jawahar Medical Foundation's Annasaheb Chudaman Patil Memorial Dental College, Dhule, IND

**Keywords:** dental implants, digital, edentulism, partial, prosthodontics, workflow

## Abstract

This case report presents the comprehensive digital rehabilitation of a partially edentulous 53-year-old female patient with multiple missing and compromised teeth, leading to impaired masticatory function and esthetics. A multidisciplinary treatment approach was employed, incorporating advanced diagnostic, surgical, and prosthetic protocols, to achieve predictable and patient-centered outcomes. Initial clinical and radiographic evaluations, including cone-beam computed tomography, facilitated accurate assessment of bone availability and guided the treatment plan. This case involved staged extractions, endodontic treatment, periodontal care, and strategic implant placement in both the maxilla and mandible. In the posterior maxilla, sinus augmentation was performed to overcome the limited bone height and enable successful implant placement. Digital planning tools have been used to design a virtual prosthetic outcome, enhancing precision and patient communication. A custom anterior deprogrammer was used to establish a stable occlusal relationship. Following osseointegration, intraoral scanning and computer-aided design/computer-aided manufacturing technologies were used to fabricate posterior metal-ceramic and anterior zirconia restorations, ensuring high accuracy and esthetic integration. Postoperative care included comprehensive oral hygiene instructions, dietary modifications, and structured follow-up visits. At the three-month review, the patient demonstrated excellent peri-implant tissue health, functional occlusion, and high satisfaction with the esthetic results. This case highlights the value of combining digital technologies with a multidisciplinary clinical approach for the management of complex implant rehabilitation. This outcome emphasizes the importance of precision, planning, and patient education in achieving long-term success in implant-supported prosthodontics.

## Introduction

Tooth loss due to caries, periodontal disease, or trauma can significantly impair a patient’s ability to chew, speak, and maintain oral esthetics, ultimately affecting their quality of life [1]. The evolution of dental implantology has significantly transformed the management of partial and complete edentulism, offering patients reliable, long-term solutions that restore both function and esthetics [2]. Dental implants have become the cornerstone of modern prosthetic dentistry because of their high success rates, excellent biocompatibility, and ability to preserve the alveolar bone [3]. Their predictability and integration with surrounding tissues make them particularly advantageous for complex restorative cases [4].

In recent years, the integration of advanced digital technologies has further enhanced the precision and efficiency of implant planning and placement [5]. Tools such as cone-beam computed tomography (CBCT), intraoral scanners, and computer-aided design/computer-aided manufacturing (CAD/CAM) systems enable clinicians to adopt a fully digital workflow [5,6]. This approach facilitates accurate diagnosis, optimal prosthetic design, and minimally invasive procedures, ultimately leading to improved patient outcomes and satisfaction.

This case report describes the multidisciplinary rehabilitation of a 53-year-old female patient who presented with multiple missing and compromised teeth, resulting in functional limitations, particularly impaired mastication. Clinical and radiographic evaluations revealed the need for extensive prosthodontic and surgical intervention. A detailed treatment plan involving multiple extractions, endodontic therapy, periodontal care, and staged implant placement was developed. Digital tools play a pivotal role in diagnostics, implant planning, mock-up visualization, and final prosthesis fabrication. By integrating advanced technologies with a phased clinical approach, this case exemplifies the potential of contemporary implant dentistry to address complex full-arch rehabilitation and restore both function and esthetics in partially edentulous patients.

## Case presentation

**Figure 1:**
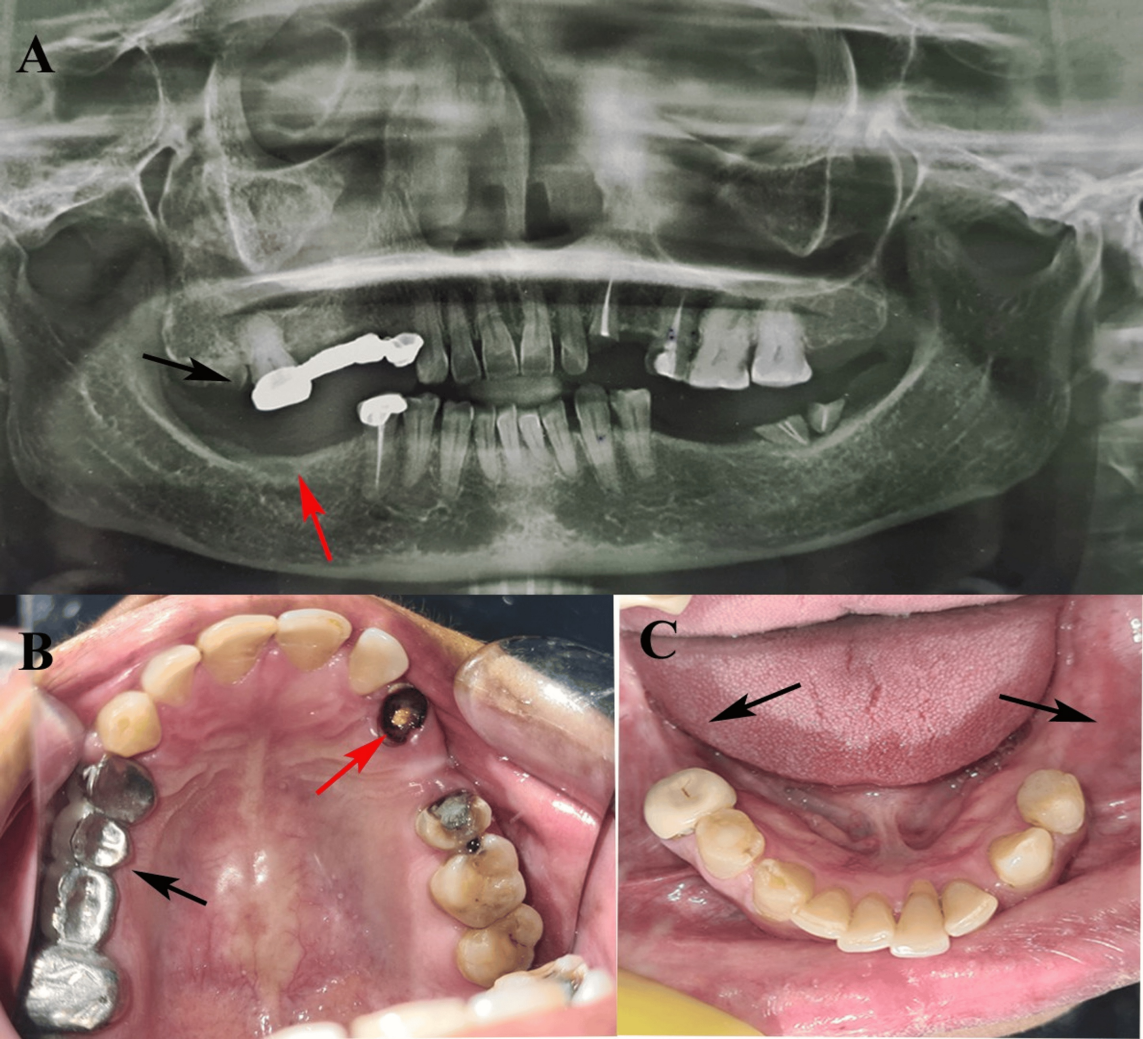
Preoperative features. (A) CBCT showing a metal bridge in the maxilla (black arrow) and an edentulous area in the mandible (red arrow). (B) Maxillary jaw showing a metal bridge on the right side (black arrow) and a root canal–treated root remnant of the canine on the left side (red arrow). (C) Mandibular jaw showing bilateral posterior edentulous areas (black arrows). These images are related to our patient and published with the patient's consent. CBCT: cone-beam computed tomography.

A 53-year-old female patient presented to the Department of Prosthodontics, Jawahar Medical Foundation's Annasaheb Chudaman Patil Memorial Dental College, Dhule, India, in January 2024 with a chief complaint of difficulty chewing due to multiple missing teeth. Informed patient consent for publication has been obtained, with no identifiable information disclosed and confidentiality maintained. Her medical history was non-contributory, and laboratory findings of blood investigations were within normal limits. Intraoral examination revealed a metal bridge in the upper right posterior region from the first premolar to the second molar (14-17), a metal ceramic crown on the lower right second premolar (45), and root canal treated root remnants in the upper right third molar (18), upper left canine (23), and lower left third molar (38). The upper left first premolar (24) was missing. Mild pathological migration was noted in the upper anterior teeth, along with caries on the distal surface of the upper left first molar (26), and Grade II mobility of the lower left central incisor (31) was present. Oral hygiene condition was suboptimal (Figure 1).

Diagnostic impressions were recorded, followed by panoramic radiography and CBCT scans using a Dentium CBCT unit (Dentium Co., Ltd., Seoul, South Korea). Interdisciplinary evaluations, including vitality testing, periodontal assessment, and oral prophylaxis, were performed. CBCT revealed insufficient bone height in the region of the upper right first molar (16), necessitating sinus lift. A phased multidisciplinary treatment plan combining endodontic therapy, surgical intervention, and prosthetic rehabilitation was formulated.

**Figure 2:**
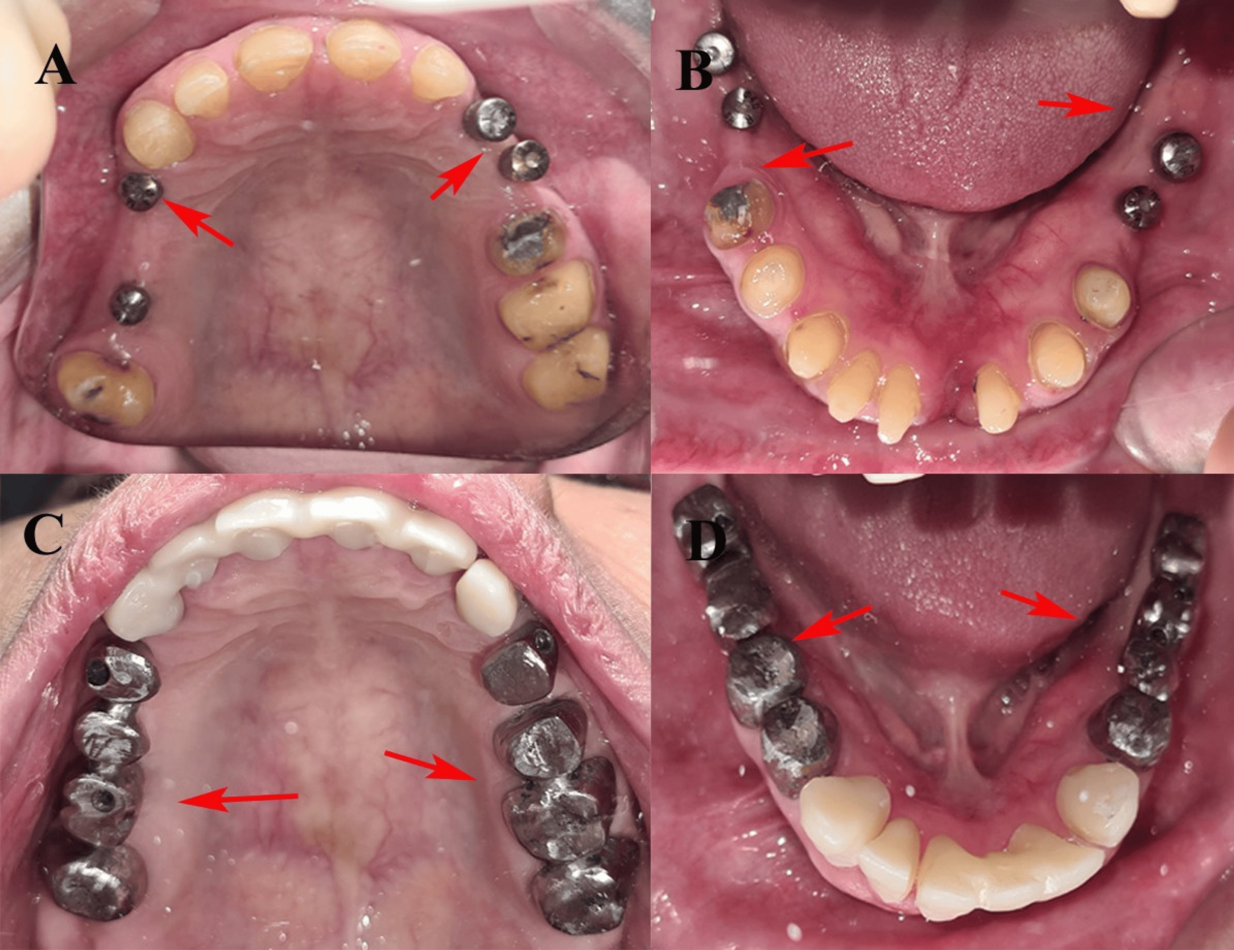
Post-implant placement. (A) Maxillary jaw with implants placed on the right and left sides with abutments. (B) Mandibular jaw with implants placed on the right and left sides with abutments. (C) Maxillary jaw showing metal try-in. (D) Mandibular jaw showing metal try-in. These images are related to our patient and published with patient's consent.

Root canal treatment was completed in the lower left and lower right first premolar teeth (34 and 44). The lower left central incisor (31), lower left third molar (38), and upper left canine (23) root remnants were extracted because of poor prognosis. A diagnostic wax-up (digital) preceded implant placement to plan occlusion, esthetics, and prosthetic space. In the first surgical phase, Osstem TSIII dental implants (Osstem Implant Co., Ltd., Seoul, South Korea) were placed in the lower right first and second molars (46 and 47, respectively), lower left second premolar (35), and lower left second molar (37). In the second phase, after extracting the upper right third molar (18) and upper left canine (23), implants were placed in the upper left canine (23), upper left first premolar (24), and upper right first premolar (14). An indirect sinus lift procedure was performed at the site of the upper right first molar (16), and 0.25 cc of NovaBone putty (NovaBone Products, LLC, Alachua, Florida) was used for grafting before implant placement (Figure 2).

Following implant placement, the patient was provided detailed postoperative instructions to ensure proper healing and long-term implant success. She was advised to avoid hard or chewy food over the surgical sites during the initial healing phase and to maintain a soft diet for at least one week. Cold compresses were recommended to reduce postoperative swelling, and the patient was prescribed a course of antibiotics and analgesics. A chlorhexidine mouthwash (0.12%) was advised twice daily for one week to maintain oral hygiene without disturbing the surgical area. She was instructed not to brush the surgical sites directly for the first few days and to avoid smoking or alcohol consumption, which could interfere with healing.

**Figure 3:**
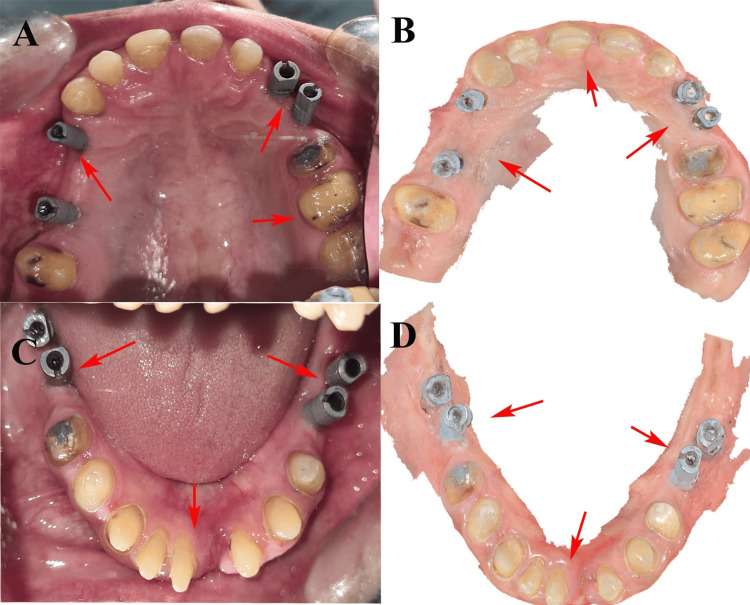
(A) Maxillary jaw with impression coping. (B) Digital impression of the maxillary jaw. (C) Mandibular jaw with impression coping. (D) Digital impression of the mandibular jaw. These images are related to our patient and published with patient's consent.

To deprogram occlusal interference and establish a stable centric relation, a custom anterior bite plane deprogrammer was fabricated using DPI autopolymerizing acrylic resin (Dental Products of India, Mumbai, India). A digital mock-up of the proposed restoration was created using the Exocad CAD software (Exocad GmbH, Darmstadt, Germany), enabling accurate prosthetic planning and patient visualization. Three months after implant placement, second-stage surgery and prosthetic tooth preparation were performed (Figure 3).

**Figure 4:**
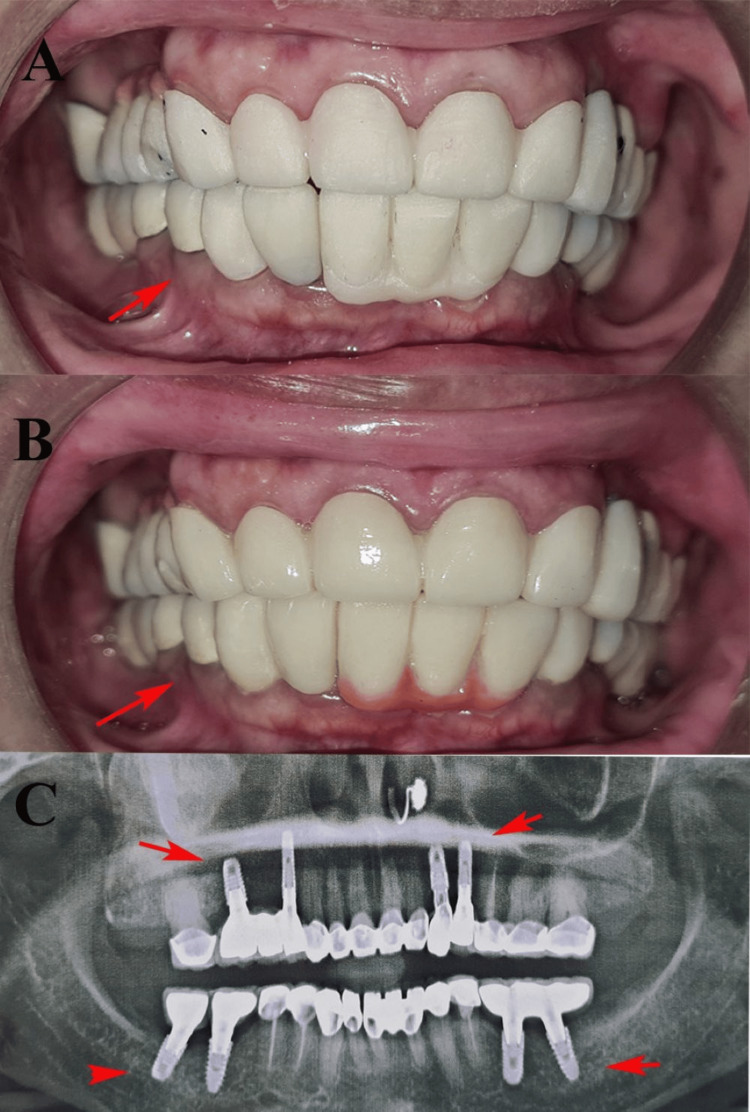
(A) Bisque try-in for maxillary and mandibular teeth. (B) Polished metal-ceramic maxillary and mandibular implant-supported hybrid bridge. (C) Postoperative follow-up orthopantomogram (OPG) after three months. These images are related to our patient and published with the patient's consent.

Intraoral scanning was performed using a Dexis intraoral scanner (Dexis, Quakertown, Pennsylvania, USA). Metal copings for the posterior prostheses were fabricated using a D-100 metal 3D printer (Dentis Co., Ltd., Daegu, South Korea), and zirconia prostheses for the anterior region were milled using an ARUM Dentistry SX-300 Pro milling unit (ARUM Dentistry Co., Ltd., Daejeon, South Korea). A bisque trial was conducted to verify the aesthetics, fit, and occlusal harmony (Figure 4).

After the second-stage surgery and prosthetic loading, the patient was instructed to maintain meticulous oral hygiene around the implants by using a soft-bristled toothbrush, interdental brushes, and floss. The importance of regular follow-up visits and professional maintenance therapy has been emphasized in monitoring peri-implant tissues and prosthetic components.

The final prosthesis was delivered under mutually protected occlusion and canine guidance. The patient was recalled one week, one month, and three months after post-prosthesis delivery. At the three-month follow-up, clinical and radiographic assessments revealed stable implant integration, healthy soft tissue, and satisfactory occlusion. No signs of inflammation, mobility, or prosthetic complications were noted. The patient reported significant improvements in mastication and esthetics, with high satisfaction regarding treatment outcomes (Figure 4). This case demonstrates the effectiveness of integrating digital workflow and multidisciplinary care in the successful rehabilitation of a partially edentulous patient.

## Discussion

Successful rehabilitation of partially edentulous patients with dental implants relies heavily on precise planning, interdisciplinary coordination, and long-term maintenance [2]. The use of a digitally integrated workflow facilitated by CBCT imaging, intraoral scanning, and CAD/CAM technology contributed to predictable and patient-centered outcomes.

The selection of Osstem TSIII implants, known for their enhanced surface topography and high primary stability, enables secure placement even in anatomically compromised regions. Kim et al. [7] reported a 95.37% seven-year cumulative survival success rate. Insufficient bone height is a significant determinant of implant placement in the posterior maxilla [8]. The loss of dentition may culminate in a deficiency of functional loading, which can precipitate considerable bone resorption, thereby resulting in attenuation of the bony walls and subsequently facilitating pneumatization of the sinus [9]. This phenomenon engenders a compromise in prospective implant placement and necessitates supplementary surgical interventions for bone augmentation [10]. In the present case, particularly in the posterior maxilla, where limited bone height is often a concern, the use of indirect sinus lift with NovaBone putty provided adequate augmentation to allow successful implant placement.

The use of digital planning software and intraoral scanners has significantly transformed implant prosthodontics [5]. Exocad-based digital mock-ups enabled the pre-visualization of esthetic outcomes, while intraoral scanning enhanced the accuracy and efficiency of impression procedures. Papaspyridakos et al. [11] described a similar treatment in three patients where a fully digital workflow was used in a fully edentulous patient to achieve highly accurate full-arch implant rehabilitation, demonstrating excellent prosthetic fit, patient satisfaction, and 100% success rate at two-year follow-up. Another study by Joda and Gallucci [12] supported the clinical relevance of the "virtual patient" approach in prosthetic planning, citing its advantages in terms of precision and efficiency.

Furthermore, the use of 3D-printed metal copings and CAD-milled zirconia restorations allows for high customization and durability. Lang and Berglundh [13] emphasized that long-term implant success is closely linked not only to precise surgical and prosthetic protocols but also to the maintenance of healthy peri-implant tissues through patient compliance and structured follow-up.

The three-month post-treatment follow-up in this case revealed stable integration of all implants, absence of complications, and favorable esthetic and functional results. The implementation of digital workflows presents a significant opportunity to not only reduce the duration of rehabilitation processes but also enhance the overall satisfaction levels of patients, a phenomenon that can be attributed to the inherently less invasive characteristics associated with such modern methodologies [14,15].

This case underscores the value of combining surgical expertise, restorative planning, and digital innovations to manage complex partially edentulous scenarios. Digital dentistry not only streamlines workflow but also improves predictability, prosthetic fit, and overall patient experience. This case highlights the importance of a multidisciplinary approach that integrates endodontic, periodontal, and prosthodontic expertise to achieve a comprehensive rehabilitation.

## Conclusions

This case report illustrated successful multidisciplinary rehabilitation of a partially edentulous patient using a fully digital workflow and implant-supported prostheses. The integration of advanced diagnostic imaging, CAD/CAM planning, and 3D printing technologies enabled precise implant placement and prosthetic fabrication, resulting in optimal esthetic and functional outcomes. Careful case selection, staged treatment, and patient education are critical for achieving stable results. This case underscores the transformative impact of digital dentistry in managing complex implant cases and highlights the importance of collaborative patient-centered care for long-term success.
